# Wanting More, Seeing Less: Hunger Reduces Calorie Evaluations

**DOI:** 10.3390/ijerph182312270

**Published:** 2021-11-23

**Authors:** Aner Tal

**Affiliations:** College of Law and Business, Ramat Gan 52573, Israel; anert@clb.ac.il

**Keywords:** food, calorie estimation, dieting, hunger, portion size, motivated reasoning, functional judgment, nutrition literacy

## Abstract

Calorie estimates play an important role in the regulation of food consumption. Lower calorie estimates contribute to increased consumption, and consequently increase the risk of obesity. The current work presents a novel contribution demonstrating the biasing effect of hunger on calorie evaluations. Study 1 (N = 70) was a field study, where participants visiting a cafeteria estimated calories in four baked goods, with measures taken of their hunger level and their having had lunch. Study 2 was a lab study, where half of the participants (N = 65) fasted for five hours prior to the study, and then estimated calories in three baked goods. Study 1 found lower calorie estimations by hungry participants (M = 255.52, SD = 112.55) relative to lower hunger participants (M = 311.94, SD = 135.85): F(1, 67) = 6.07, *p* = 0.02. In study 2, the average estimated calories was lower for fasting participants (M = 253.11, SD = 126.13) than for non-fasting participants (M = 301.75, SD = 145.26). The studies demonstrate that motivations generated by physical state (hunger) alter calorie evaluations. This finding is surprising given that motivation generally leads to estimating more of a desired quantity. The study also presents a novel domain of biases in calorie estimation. This reduction in calorie estimates due to hunger may occur if calories are assessed relative to needs, or serve to allow people to justify increased food consumption. Accounting for such biases, particularly in cases of low nutrition literacy, is important in order to reduce the overeating that may be generated by calorie estimation biases.

## 1. Introduction

Portion size estimation constitutes a large source of error in dietary recalls [1]. People consistently underestimate portion sizes when it comes to physical size, weight, and calories, particularly of large servings [2]. Portion size and calorie estimation are closely related, as bigger sizes are naturally associated with increased calories.

Though much research has examined consumer biases in calorie estimation, little research exists that examines the effects of the physical state on calorie estimations. Accordingly, the current research aims to offer novel findings regarding the impact of physical state on calorie estimation. Specifically, it examines how hunger, a high motivational state, reduces estimation of calories.

Calorie estimates can be of high importance to food choices, the monitoring of consumption, and weight management [3,4]. Biases in portion size and calorie estimation may make regulation of calorie consumption more difficult. Perceiving a product as less caloric can encourage consumers to overeat [5,6]. Increased consumption due to lower estimates can, in turn, contribute to obesity [7,8,9]. Those most prone to calorie underestimations tend to have increased body mass, indicating a connection between underestimation of calories and overweight, though it is unknown whether high BMI causes lower estimation [10,11,12]. Overweight is in turn associated with many health problems and increased mortality [13,14]. All this highlights the importance of calorie estimates for public health.

In the current work, I examine how a common physical state, hunger, influences calorie estimation. Given the prevalence of hunger, such an effect is common and highly relevant for consumers’ purchase decisions [15,16,17]. In addition to its practical importance, the effect of hunger on calorie estimation also offers an interesting case of motivated judgment and reasoning, the judgment of reality according to the way one desires it to be. Notably, most motivated reasoning studies demonstrate higher estimations of desired quantities and objects [18,19].

### 1.1. Biases in Calorie Estimation

Calorie estimation may be inaccurate due to low nutritional literacy and lack of sufficient education. Beyond a lack of sufficient education, a wide variety of factors can bias food quantity estimates [20]. Bad mood, for example, can modulate the impact of self-control on portion size estimation [21], as well as leading to disinhibited eating and overestimation of consumption amounts [21,22,23,24]. Additional factors, such as product dimensionality, can also bias evaluations of serving size, and consequently affect meal size [25].

Consumers’ calorie estimates are particularly prone to distortions. Calorie estimates are often inaccurate [6,26,27]. Most studies find an underestimation of calories [27,28,29,30,31]. These effects have been demonstrated in surveys, in the lab, and in the field [32,33]. Underestimations may be worse for overweight people [10,34], whereas dieters tend to have greater accuracy [35]. Notably, some studies demonstrate an overestimation, rather than an underestimation, of calories [10,11,36].

Categorization of foods as healthy and unhealthy can play a large part in driving calorie underestimation. Foods commonly regarded as unhealthy are often viewed as having more calories than foods perceived as healthy [37]. Consumers may underestimate the calories in healthy foods due to their health halos, while overestimating calories in foods perceived as unhealthy [34,36]. Wansink et al. (2006) have also shown that elements which encourage a healthy image, such as “low-fat” nutrition claims, lead to reduced calorie estimation [5]. Calorie estimates are also reduced when foods come from restaurants that are seen as healthier [6].

The effects of health perceptions on calorie estimation can result in an ironic effect, whereby adding a healthy item to an unhealthy item can result in lowered calorie evaluation for the two items in combination [38]. Categorization of foods as healthy or unhealthy can also bias sequential calorie evaluation, with food seen as healthy (unhealthy) being evaluated as less (more) caloric when following evaluation of an item from the opposite category [39].

Table 1 below summarizes some of the factors documented as affecting calorie estimation.

### 1.2. Hunger and Calorie Estimation: Would Hunger Lead to Increased Calorie Estimates?

Research on motivated estimation suggests that hunger may lead to estimation of greater calories. Bruner and Goodman (1947) first investigated the effect of value and need on size perception. In their experiment, coin sizes were estimated as larger by poorer children, with higher value coins judged as larger. Their theoretical account was that when need for an object is great, it is seen as larger [40].

Though the Bruner and Goodman studies have been heavily criticized [41,42], subsequent work has provided fresh evidence for similar motivated estimation effects, where needs make objects appear larger. Findings have emerged demonstrating that people tend to see what they want to see [43]. When people desire an object, the object appears larger and closer [44]. Accordingly, distances are seen as shorter when one wants them to be short [45]. Similarly, garden tools are seen as larger when one wants to garden [19]. Overall, people see desired objects as larger.

A popular explanation for this phenomenon rests on functional notions. Generally, perception works in the service of action, such that people perceive the environment in ways that help need fulfillment [46]. Specifically, objects that are needed loom larger perceptually so as to draw attention and prompt action to obtain them [18,19,44]. Such attentional salience would make objects appear closer, and consequently larger.

From this perspective, and given the relation of calories to quantity and size—bigger portions tend to contain more calories—hunger should make people estimate higher calories. Though no research paper has directly addressed this question to my knowledge, some research has examined the relation of hunger to size perception, and provided initial evidence in support of this prediction. Beasley et al. (2004) found that portion sizes were generally estimated as smaller when participants were fed, and larger when participants were hungry [47]. Similarly, palatable foods are estimated as larger by dieters vs. non-dieters [18], and water glasses are estimated as larger to people who are thirsty [19].

All this gives reason to suspect that hunger may lead to increased calorie estimates. However, calories may behave differently than size estimates. Although calories involve quantity, they are not based solely on physical size. Indeed, much of one item (watermelon) can be less caloric than little of another item (butter). In addition, unlike size, calories are not physically observable. One cannot see calories, such that they cannot “loom larger”, and hunger cannot lead a person to see more of them. These factors may lead calories to behave differently than size estimates.

Since perceptual salience would not apply to calories, which are not physically observable, it may well be that calorie estimation would not be affected by need in the same way as size estimation. In fact, hunger may, in this case, lead people to estimate foods as containing less, rather than more, calories, as I argue next.

### 1.3. Why Would Hunger Lead to Reduced Calorie Estimates?

A higher need for food, represented by a higher hunger level, leads people to feel a greater desire for food. This is reflected in a higher responsiveness to food and increased ratings of food appeal by hungry people [48]. Such enhanced attraction occurs selectively with particular foods, especially higher-calorie foods [49,50]. This leads hungry participants to show a preference towards higher-calorie foods [51]. Foods are reported to be tastier, and fattier food is seen as more appealing, and chosen more often, when one is hungry [52,53]. These changes in food judgment and evaluation can in turn lead to increased food purchases in general [15,16], and to increased purchases of higher-calorie foods in particular [17]. All these point to the impact of hunger on food desirability and consumption, particularly of high-calorie foods.

To legitimize the consumption of higher-calorie foods, consumers may be motivated to downplay the number of calories in food, particularly calorie dense food, when hungry. This is because eating high calories foods can generate feelings of guilt. Accordingly, people often seek justification for indulgent consumption [54]. Estimating a food as possessing lower calories than it actually has can provide such a justification and help legitimize increased consumption.

In support of the role of calorie evaluation in justifying consumption, prior research demonstrates that when consumers judge a food to be healthier, they allow themselves to eat a greater amount [55,56]. Similarly, thinking a food is lower in calories can allow people to justify increased consumption [5].

As argued above, hunger increases the draw towards higher calorie foods, leading participants to desire greater amounts. When desire is greater, due to hunger, people may be particularly motivated to legitimize consumption by estimating lower calories. Thus, motivated estimation in this context might mean estimation of less, rather than more, calories, when hungry. In other words, hunger may lead consumers to have greater motivation to downplay the number of calories in food, in order to legitimize, or license, increased consumption.

A second reason for hunger leading to reduced calorie estimates may rest on the functional nature of perception and judgment [46,57,58,59,60,61]. Functional judgment claims that object properties are perceived relative to needs. When hungry, calories are lower, relative to one’s needs. Given higher caloric needs, each food would supply a lower amount of calories relative to need. If calories are evaluated in a subjective sense, then they may be seen as lower when need (hunger) is higher. From a functional perspective, then, hunger may lead people to judge calories as objectively lower, since any portion supplies a lower amount of calories in relation to needs.

This reasoning would lead us to predict the opposite of the prediction outlined in the previous section: that increased hunger would lead to reduced calorie estimates. The studies presented here intend to examine whether hunger would lead to increased or decreased calorie estimates. As discussed above, in theory, either possibility may occur as a result of motivated estimation and the functional nature of perception and judgment. To whit, if calories behave similarly to size estimates, and loom larger when hungry, hunger should lead to increased calorie estimates. If, on the other hand, calories behave differently than size, people may be motivated to estimate lower calories when hungry to license increased eating, such that hunger would lead to reduced calorie estimates.

The studies below are intended to test which of the two competing hypotheses outlined in these two sections holds:

**H_1a_**: Increased hunger would increase calorie estimation.

**H_1b_**: Increased hunger would reduce calorie estimation.

## 2. Study 1 Materials and Methods

In this IRB-approved study, 70 participants (mean age 24.04 years, SD = 8.55, 32 female) were randomly approached outside a cafeteria at a large Northeastern university around lunchtime. Note that the study was conducted prior to the COVID-19 pandemic. Participants were asked to participate in a short survey in exchange for a small token of thanks (dollar bill or snack bar). Those who agreed gave informed consent for participation. Participants included undergraduate students, graduate students, and university employees.

Participants completed the study in small groups, in the presence of the researchers, who continued recruiting participants as current participants completed the study. No participants were excluded from the study. All interested participants were allowed to participate. The cafeteria setting was chosen as a realistic field setting which draws a diverse population. The setting also helped maximize distribution of hunger levels, since I assumed that some participants would be recruited before lunch, and some after, with pre-(post) lunch participants leading to a higher (lower) hunger level.

Participants were presented with four baked goods (muffin, bagel, donut, and brownie). These items were chosen for the following reasons: (a) Calorie underestimation increases with calorie content, so that items with a sufficient calorie level are needed to provide an ideal setting for finding variation in calorie estimation [6]. In addition, (b) these items are familiar enough to minimize noise, but not so familiar that participants are likely to know the exact amount of calories in each, allowing for variation due to our manipulation.

All items were similarly sized. All items were presented together, and positioned a few inches from each other on paper plates. Participants were not allowed to hold the items, and knew that the items were for display only, such that they would not actually be in their possession.

The number of calories in each item was estimated in an open-ended measure. Each participant estimated calories in all items.

After estimating calories in the different foods, participants were asked to report their hunger level on a continuous Likert scale of 1 (not at all hungry) to 9 (very hungry), where participants could circle any number between 1 and 9 to designate their level of hunger. They were also asked to report whether they had eaten lunch yet or not (binary).

## 3. Study 1 Results

The statistical tests were designed to examine the effects of hunger on calorie estimation. Data were analyzed using a repeated measures mixed model, with the item type as the repeated factor. Item type was included in the model as a covariate. The covariance matrix was specified as unstructured, to allow for a maximally conservative test involving minimal assumptions. Two models were conducted for the two alternative independent variables, one with having had lunch as a binary measure, and the second with hunger as a continuous measure.

The hungrier participants were, the lower the number of calories estimated: F(1, 67) = 6.07, *p* = 0.02. While the model itself used hunger as a continuous measure, a median split on hunger was conducted to illustrate the size of effects. Examining calorie means across low and high hunger levels, high hunger participants estimated a lower average number of calories (M = 255.52 kcal, SD = 112.55) than did low hunger participants (M = 311.94 kcal, SD = 135.85), with a difference of 22%.

Next, whether the effects were robust to the type of food item was examined. This was done by including the food item type as an element in the model as both a main effect and an interaction effect with hunger. Examining an interaction of the item and hunger revealed that the effect was robust across items. In other words, there was no significant interaction between hunger and item type: F(3, 67) = 0.26, *p* = 0.86.

A second model looking at the binary variable of lunch eaten, and as an alternative, the behavioral operationalization of hunger, was run. Using the binary measure of whether participants have eaten lunch yet (n = 33) or not (n = 37) produced a result similar to that revealed using hunger as an independent variable. Participants who had not yet eaten lunch (hungry) reported 16% less calories (M = 263.87 kcal, SD = 128.67) than those who had already eaten lunch (M = 305.99 kcal, SD = 123.3): F(1, 68) = 4.4, 0.04.

Finally, the robustness of the “lunch” model to the food item type was examined. This was done using a model of the effect of having had lunch on calorie estimation that included the item type and its interaction with having had lunch. There were no significant differences between items: the interaction was not significant: F(3, 67) = 0.26, *p* > 0.1. In other words, the effect of having had lunch on calorie estimation was robust across food item types. The calorie means for those who have or have not eaten lunch for each item are displayed in Table 2 below.

Table 1 also displays approximate objective calories for each of the items, to allow for a comparison between objective calories and participants’ estimates. Notably, participants underestimated calories across all food items and almost all cells, consistent with prior research [10,11,36].

Notably, the underestimation of calories was markedly higher for those who estimated calories while hungry. For example, while post-lunch participants underestimated the calories in a bagel by approximately 5% (15 calories), for those who estimated calories before lunch, the underestimation was approximately 24% (73 calories). For brownies, while after-lunch participants underestimated calories by 19% (78 calories), pre-lunch participants underestimated calories by 29% (115 calories). For muffins, there was an underestimation of 17% (67 calories) for post-lunch participants, vs. 23% (92 calories) for pre-lunch participants. For donuts, post-lunch participants actually overestimated calories by approximately 13% (33 calories), while pre-lunch participants underestimated by 6% (16 calories). Across items, while underestimation was approximately 9% (32 calories) for those who estimated calories after lunch, it was approximately 2.5 times that, 22% (74 calories), for those who estimated calories before lunch.

In conclusion, the study demonstrates that higher hunger levels lead to reduced calorie estimates, as well as increased underestimation of calories. In this, the study confirms H_1b_. In the next study, I aimed to find whether random assignment to hunger manipulation conditions would produce similar effects, helping preclude potential self-selection as a cause for the findings of the first study.

## 4. Study 2 Materials and Methods

The first study relied on natural variation in hunger levels, and so cannot be used to conclude that hunger causes a reduction in estimated calories. In our main study, I manipulated hunger by asking half of our participants (N = 65 total sample, mean age 19.72 years, SD = 2.64, 22 female) to avoid eating for five hours prior to the study. This study was also conducted prior to the COVID-19 pandemic.

Participants were undergraduate students recruited from the participant pool of our behavioral lab at a large Northeastern University, and completed the study for credit. No participants were excluded from the study. Sample size was determined based on a rule of thumb from previous research determining approximately 30 per cell to be the appropriate cell size for this type of study (e.g., [19]). Participants signed up for one of two days. One of the days was arbitrarily determined by the experimenters to be the fasting condition day, and the other the non-fasting condition day. Participants were not informed of condition in recruitment. Both participant groups completed the study on a Wednesday, but a week apart. The two runs were completed exactly a week apart to control for day-of-the-week variations while minimizing other temporal variation. The study was run during similar hours on both days, between 11:00 a.m. and 5:00 p.m. We did not give a reason for fasting.

Participants completed the study for credit. The research team obtained informed consent from each participants for this IRB-approved study. Participants completed the study in our behavioral lab, where the research team displayed three items: a muffin, bagel, and giant cookie. I chose these foods to mirror, and replicate, our previous study, which featured similar baked goods. The three baked goods were displayed side by side on a table, with participants visually inspecting items, without touching, and estimating the number of calories in each baked good on an open measure. As before, participants knew that they would not taste the displayed items. Participants also reported on their hunger level using the same scale of 1–9 employed in the last study, using a continuous Likert scale anchored by 1 (=not at all hungry) to 9 (=very hungry). To further verify adherence to fasting, the questionnaires also asked participants to report the amount of time elapsed since the last time they had eaten prior to the study.

## 5. Study 2 Results and Discussion

First, the effects of the manipulation were validated by running a manipulation check on the effects of fasting on hunger levels. Fasting had a significant effect on hunger level: t(62) = −4.01, *p* = 0.0002. The hunger level was 4.11 (SD = 1.85) for non-fasters, and 6.14 (SD = 2.1) for fasters. The reported time elapsed since last meal was 8.91 h (SD = 3.84) for fasting condition participants, and 2.97 h (SD = 3.4) for no-fasting condition participants. The difference was significant at a <0.0001 level, t(56) = −6.07. This indicates that the manipulation was indeed effective at prompting fasting.

Out of 30 participants assigned to the fasting condition, three participants did not report on the duration of time they had not eaten. Only four participants reported having eaten less than 5 h prior to the study, and only one reported having eaten less than 4 h prior to the study. Out of 28 participants assigned to the non-fasting condition, four participants did not report on the duration of time during which they had not eaten. Only one participant had not eaten for more than 5 h prior to the study.

In sum, fasting instructions produced significant differences in hunger and time since the last meal. Participants who did not report on time since their last meal were not eliminated, to avoid undoing the random assignment to conditions, as well as reducing our sample size. Accordingly, regardless of their report on fasting, participants from the analysis were not excluded. Five participants were excluded from analysis due to missing values on calorie estimations, that is, not fully reporting the dependent variables.

Analysis was conducted using the assigned condition (fasting/no fasting), regardless of adherence. This was done to conserve random assignment and due to the ability to draw causal conclusions from the study.

The effects of fasting on calorie estimation were examined using a repeated measures mixed model with the food item as the repeated factor. As in the previous study, the covariance matrix was specified as unstructured. The independent variable was the assigned fasting condition.

There was a significant effect of fasting on calorie estimation: F(1, 58) = 4.61, *p* = 0.04. Average estimated calories were lower for fasting participants (M = 253.11 kcal, SD = 126.13) than for non-fasting participants (M = 301.75 kcal, SD = 145.26), a difference of 19%.

Finally, the robustness of the effects to the food item type was examined. In this case, effects of fasting on calories did vary among the different food items. Including an interaction between fasting and item type in the model produced a significant interaction: F(2, 58) = 4.75, *p* = 0.01. Specifically, the effects weakened with repeated measures, such that the later the calorie estimation, the lower the effects. Accordingly, effects were lowest for the third item (cookie). Directionally, however, all items were affected in a similar manner. That is, for all items, fasting decreased calorie estimation, as can be seen in Table 3 below.

Table 3 presents a comparison of estimated and actual calories, to provide a picture of underestimation of calories by participants relative to objective calories in the food items. As in the previous study, we see a consistent underestimation of calories across items and conditions. Similarly, as before, we see considerably higher underestimations of calories in the hunger condition. The average calorie underestimation across items was approximately 9% (32 calories) for the no-fasting condition, and approximately 24% (80 calories) for the fasting condition. For individual items, approximate underestimation increased from 11% (43 calories) to 34% (136 calories) for muffins, from 14% (41 calories) to 28% (85 calories) for bagels, and from 4% (11 calories) to 7% (20 calories) for cookies.

Study 2, then, replicated the effects of the first study, supporting H_1b_, while manipulating (rather than measuring) hunger. Participants who evaluated food when hungrier estimated a lower number of calories in the food.

## 6. General Discussion

Two studies, in field and lab settings, with both naturally varying and manipulated hunger, demonstrated that increased hunger leads to reduced calorie estimates. The findings highlight that at least under some circumstances, needs can lead people to perceive less of a desired product property rather than more of it. This may be because estimating lower calories justifies increased consumption, and because calories are estimated relative to one’s needs, which are higher when hungry. Accordingly, reducing calorie estimates when hungry can be seen as a type of motivated reasoning.

The studies contribute to current knowledge in several respects. First, these findings may explain prior results showing that hunger leads people to purchase more, and higher-calorie, groceries [15,16]. The findings also contribute to our understanding of how physical states, and hunger in particular, affect product judgment. Most prior research has focused on the effects of hunger on preference and choice. The current studies demonstrate that hunger can have systematic effects on calorie evaluation, an important factor in portion control and dietary regulation. The findings also offer specific novel insights into calorie estimation, suggesting that need states may systematically alter calorie evaluations [26]. On the applied side, these findings are of importance for public health, in that lower-calorie estimates may lead to overconsumption [6]. Some of the contributions are elaborated on below.

In the current studies, foods were perceived as offering lower estimated calories, rather than higher calories, when a motivation to eat was present (hunger). The studies are thus important for our understanding of how motivational states affect judgment. They present an interesting case whereby a motivational state (hunger) leads people to estimate less, rather than more, of a desired quantity (calories). The findings also suggest the intriguing possibility that judgment of different product attributes could be functional, contributing to theories of functional perception [46]. People often perceive their environment and objects in it in terms of how it allows them to act and fulfill needs [56,57,58,59,60]. When one needs more food (i.e., is hungrier), any given quantity of food would appear to provide less sustenance, relative to one’s needs. Calories may be estimated not absolutely, but relative to need.

### 6.1. Limitations and Future Research

The current research only explores one operationalization of a need state for food—hunger. Other need states can also lead to reduced calorie evaluation. For instance, low blood sugar levels may lead to reduced estimation of calories in high-sugar foods. Exploration of the effects of further need states can further support the theory that people estimate lower calories in foods for which they have greater need, to legitimize their consumption.

More generally, consumers may judge products not according to some objective standard, but rather according to how they satisfy current needs and allow desired actions. When my need for a particular product quality is greater, the subjective amounts of that quality supplied by the product should be seen as lower. Future studies can expand to study such effects of need on judgment.

The lack of uniform adherence to the manipulation in study 2 may contribute to the variability of our results. This joins overall high variability in calorie measurement [2] to lead to some inconsistencies in effect sizes, and lower overall effects. However, even so, average consistent differences of about 20% were found in calorie estimations across the two studies, by no means a negligible effect. Future research with tighter control and perhaps screening for calorie estimation capabilities may produce more consistent, and stronger, effects across items.

The studies are also limited by the population included, a general college population including employees (study 1) and a student population (study 2). Future research should replicate the effects with a broader population, for greater generalizability.

Further, the studies are restricted by using particular foods—baked goods. While consistently demonstrating effects for such foods enhances the likelihood that effects are real by providing tighter control and reducing variability, future research should explore whether effects maintain across different food types, for example, foods that are not a single unit (e.g., mashed potatoes, fries) and are not high in carbohydrates (e.g., fish, meat, salad, yogurt).

It may also be interesting to examine whether similar effects occur for healthier foods. For healthy foods, justification of increased consumption when hungry may not be necessary. Hence, if the reduction in calorie estimations when hungry is indeed generated by a need to justify increased consumption, such effects may not occur for healthier foods, providing some evidence for our suggested mechanism.

The current studies focused on judgment, and did not demonstrate specific effects of calorie underestimation due to hunger on food consumption. Future research should explore whether reduced calorie estimations as demonstrated here do indeed lead to increased food consumption. Future research should also attempt to garner evidence for the process underlying these effects, testing out the motivated estimation and functional accounts as well as ruling out potential alternative explanations.

Further research can explore the justification account by exploring people’s sense of justification. If motivated judgment and a desire to legitimize increased consumption are indeed behind the results, we should see an increased feeling that consuming a food is acceptable, and reduced feelings of guilt, in cases where hunger is associated with reduced calorie estimates. Those in greater need of justification (for instance dieters, restricted eaters) should also display greater effects of hunger on calorie estimates.

In addition, research may examine other potential evidence for the mechanism behind reduced calorie estimation generated by hunger. For example, research may examine whether increased hunger leads people to perceive food as not only lower in calories, but smaller, in accordance with prior research on motivated perception.

Exploring the effects of further need states can provide support for the functional account. For example, if judgment of product qualities, such as calories, is indeed functional, we should see similar alterations due to physical states other than hunger. For instance, exercise and weight carried may also generate higher caloric needs, and so should lower calorie estimates. This would support the idea that calories are estimated relative to need, ruling out the alternative that the current effects are unique to hunger.

In this context, recent research has indicated that at least in some cases, higher need results in inflated calorie estimation [27]. Upcoming, versus just current, needs (for instance, exercise) can also influence calorie estimates. Further, giving people higher estimates of their caloric needs should reduce estimations (counter to an anchoring prediction).

In addition, future studies may examine potential moderators for the effects of hunger on calorie estimation. It may be interesting, for example, to investigate the role dietary restriction or BMI has on determining the impact of hunger on calorie estimation. It may be, for instance, that higher BMI or dietary restriction consumers are more motivated to downplay the calories in their foods when hungry, such that they would demonstrate larger effects. Such findings would help shed light on the process behind these effects, providing some support for a motivated reasoning account.

The current findings also focused on one judgment dimension only—calories. Further research can examine whether increased need also leads to lower estimates for other undesirable food dimensions (e.g., fat), to legitimize consumption. This would not only generalize the effects for additional judgment dimensions, but provide support for justification as an explanatory account for our findings.

### 6.2. Advice to Health Practitioners

The underestimation of calories that results from hunger could potentially lead to increased consumption and hamper monitoring of caloric consumption. Practitioners may want to emphasize the importance of relying on objective calorie information in these contexts and warn against making food judgments and decisions based on subjective impressions when hungry.

Currently, avoiding shopping while hungry is common advice to consumers. Here we see that not only shopping, but immediate food decisions may be adversely affected by hunger. Specifically, hunger may bias calorie estimation, and consequently consumer decision-making, leading to a choice of higher-calorie foods. Making food choices outside times of hunger, or reducing hunger while making any food choices, may be advisable. This applies to both shopping contexts and immediate meal contexts.

One piece of advice that health practitioners can give consumers as an alternative to categorically avoiding food decision-making while hungry is to curb appetites by having some food prior to decision-making that relies on calorie estimation. This may be more realistic than advising consumers to categorically avoid making food decisions while hungry, as meal choices often naturally occur in situations where consumers are hungry. Planning meals and shopping lists in advance can also help consumers avoid the biases of hunger on product judgment.

Our findings could be particularly important for dieters, for whom calories are relevant to monitor caloric consumption. Ironically, since they more often find themselves in a state of hunger caused by actual or subjective deprivation, dieters may be particularly vulnerable to reduced calorie evaluations that may hurt their attempts at energy consumption. The findings thus emphasize the danger of excessive deprivation.

## 7. Conclusions

The studies demonstrate that higher hunger levels reduce calorie estimations. More generally, they point to the role of consumers’ physiological states and needs at the time of nutrition evaluation in determining calorie estimates. In this, they provide a testament to a novel bias in calorie estimates, and point to the importance of considering such factors as consumers’ physical state at the time of nutritional assessment. Further, the studies emphasize how factors that are extraneous to the product itself, such as the consumer or their environment, may bias calorie assessment. In this, the studies underline the importance of providing objective calorie information to help combat such biases, as well as the importance of developing ways to combat particular individual and environmentally generated biases. This would be particularly important for consumers with lower nutritional literacy.

## Figures and Tables

**Table 1:** Factors affecting calorie estimates.

Factor	Nature of Effect
Low nutritional literacy	Lower knowledge about calories reduces the accuracy of estimates.
Bad mood	Bad mood leads to overestimation of calories
Products’ physical dimensions	Longer containers seen to contain less calories
Personal differences: Weight	Reduced accuracy in calorie estimation for overweight people
Perception of food as healthier	Foods perceived as healthier are evaluated as lower in calories
Hunger	Hungry people estimate lower calories

**Table 2:** Estimated Calories are Lower Before (vs. After) Lunch (N = 70).

Food	Pre Lunch Est. Kcal Mean (SD)	After Lunch Est. Kcal Mean (SD)	Approximate Objective Calories
Muffin	308.38 (136.19)	333.03 (142.23)	400
Bagel	227.43 (113.56)	285.36 (112.58)	300
Donut	234.15 (116.99)	283.15 (116.99)	250
Brownie	284.73 (105.62)	322.42 (116.78)	400

**Table 3:** Estimated Calories are Lower for fasting (vs. non fasting) condition (N = 65).

Condition	Fasting Est. kcal Mean (SD)	Not-Fasting Est. kcal Mean (SD)	Approximate Objective Calories
Muffin	264.64 (109.78)	366.66 (150.87)	400
Bagel	217.64 (121.17)	265.37 (143.72)	300
Cookie	283.14 (142.01)	291.93 (120.68)	300

## Data Availability

Data for the studies can be obtained from the author.
